# Cholera in New Orleans

**Published:** 1855-09

**Authors:** 


*Cholera in New Orleans.*—The following extract of a communication by a physician, in the New Orleans Medical News, represents the prevalence of cholera in that city as much more extensive than we were led to suppose by the action of the city government alluded to in this Journal last week:

"Notwithstanding the fact that some of our daily newspapers are constantly proclaiming our city in the enjoyment of almost unprecedented health, all who do not wilfully close their eyes and ears to existing facts, *must* be aware that the cholera is in our midst, and is doing its sad work from one end of the city to the other; it is not true that 'the cases are principally confined to the upper and lower portions of the city, and are mostly attributed to the drought, which forces a change from rain water to the meagre supply by the hydrants;' the disease is to be found everywhere throughout the city, and although it seems to attack more children and negroes, still it is to be found amongst high and low. Editors of newspapers may attempt to deceive the people in the country, and, by their strangely mistaken policy, may succeed in throwing the unwary of our population off their guard, but the sad experience of every hour in the day teaches the mass of our citizens but too truly that the cholera is in our midst, and in all its strength."

*Hospital for Women in New York.*—A meeting of the friends and promoters of this new Institution, in Madison Avenue, was held on June 2d. There were present several distinguished clergymen and members of the medical profession, and nearly a hundred ladies. Addresses were made by the Rev. Dr. Francis, (Chairman,) Dr. Horace Green, Dr. Gillman, Rev. Dr. Knox, Dr. E. H. Dixon, Dr. Sims and others. The hospital has been open about a month, and contains nineteen patients.—*N. Y. Times.*

*Treatment of Nocturnal Incontinence of Urine in Children.*—Dr. Blaschko, of Freyeawalde, asserts that he has always succeeded in this infirmity by the use of a mixture of equal parts of tr. nucis vomicæ and tr. ferri acetici, in the dose of from ten to thirteen drops, twice every evening. In one case which resisted all treatment, he resorted with success to a rotary battery; the conductor, a fine copper wire, being introduced into the meatus urinarius.

Dr. Hüber, of Zurich, recommends a mixture of ex. nuis vomicæ one part, and oxyd. ferri nigri, 48 parts, made into 24 pills, of two grains each, one of which is to be taken night and morning. Naegele recommends tannin in the dose of a grain, morning and evening.—*Gazette des Hopitaux*, collected by *Boston Med. and Surg. Jour.*

